# Lactoferrin and Nanotechnology: New Insights Into Glioblastoma Therapy

**DOI:** 10.1002/cns.71070

**Published:** 2026-09-14

**Authors:** Atena Abed, Javad Amini Mahabadi

**Affiliations:** ^1^ Department of Medical Biotechnology, School of Advanced Technologies Shahrekord University of Medical Sciences Shahrekord Iran; ^2^ Anatomical Sciences Research Center, Institute for Basic Sciences Kashan University of Medical Sciences Kashan Iran

**Keywords:** drug delivery, glioblastoma, lactoferrin, low‐density lipoprotein receptor‐related protein‐1, nanocarriers

## Abstract

**Aim:**

This review aimed to explore emerging therapeutic strategies for glioblastoma multiforme (GBM) through the integration of lactoferrin (LF) and nanotechnology, emphasizing mechanisms that improve drug delivery across the blood–brain barrier (BBB).

**Methods:**

Published studies on LF‐based nanocarriers and their biological mechanisms were systematically reviewed, focusing on LF's molecular interactions, tumor‐targeting capacity, and the design of nanoscale delivery systems capable of enhancing drug bioavailability and selectivity.

**Results:**

LF, an iron‐binding glycoprotein with antimicrobial, antioxidant, and antitumor properties, can interact with low‐density lipoprotein receptor‐related protein‐1 (LRP1), enabling its transport across the BBB and preferential uptake by glioblastoma cells. Nanocarrier systems incorporating LF improved the solubility, circulation half‐life (typically 2‐ to 5‐fold enhancement compared with free drug), and therapeutic performance of chemotherapeutic agents while minimizing systemic toxicity. Studies demonstrated that LF‐based nanoparticles could induce apoptosis, inhibit tumor growth (with tumor volume reductions ranging from approximately 40% to 70% in orthotopic GBM models), and enhance radiosensitivity in GBM models.

**Conclusion:**

LF provides a promising molecular platform for targeted glioblastoma therapy. When integrated with nanocarrier technologies, it enhances therapeutic efficacy, stability, and safety of anticancer agents. The combined LF–nanocarrier approach represents a rational and innovative strategy for improving treatment outcomes in glioblastoma multiforme.

## Graphical abstract

Current glioblastoma multiforme (GBM) management primarily relies on surgery, chemotherapy, and radiotherapy; however, therapeutic efficacy remains limited due to tumor invasiveness and restricted drug penetration across the blood–brain barrier. Lactoferrin (LF), an iron‐binding glycoprotein with receptor‐mediated transport capability, has emerged as a promising nanotherapeutic agent for targeted GBM delivery. Integration of LF‐based nanocarriers with conventional treatment modalities may enhance drug bioavailability, improve tumor‐specific accumulation, and potentiate therapeutic outcomes in GBM patients.

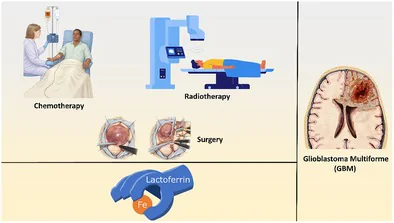

**Abbreviations**
AD: Alzheimer's disease
AIE: aggregation‐induced emission
ATR: atorvastatin
BBB: blood–brain barrier
bLF: bovine lactoferrin
BTB: blood‐tumor barrier
CRC: colorectal cancer
CS: chondroitin sulfate
DAMP: damage‐associated molecular pattern
DHA: dihydroartemisinin
DTX: deliver docetaxel
FUS: focused ultrasound
GBM: glioblastoma multiforme
GL: glycyrrhizin
GSCs: glioma stem cells
hLF: human lactoferrin
HMGB1: high‐mobility group box‐1
HSPGs: heparan sulfate proteoglycans
IDH: isocitrate dehydrogenase
IL‐6: interleukin 6
LF: lactoferrin
LF‐GL: lactoferringlycyrrhizin
LFRs: lactoferrin receptors
LRP1: low‐density lipoprotein receptor‐related protein‐1
NIMDs: nanoparticle‐in‐microparticle delivery systems
NK: natural killer
NPs: nanoparticles
PAEEP‐PLLA: poly aminoethyl ethylene phosphate and poly l‐lactide
PAEEP‐PLLA: poly aminoethyl ethylene phosphate/poly l‐lactide
PTS: pterostilbene
PTS‐SLNs: solid lipid nanoparticles containing PTS
RGD‐LF‐LP‐DTX: RGD‐LF‐modified liposomes
STAT: signal transducer and activator of transcription
TfR1: transferrin receptor 1
TME: tumor microenvironment
TMZ: temozolomide
USLP‐LF: large‐pore silica particles
WHO: World Health Organization

## 1 Introduction

Glioblastoma multiforme (GBM) represents the most prevalent and highly malignant form of brain tumor, accounting for almost 50% of all cases of diagnosed brain cancer [1]. This malignancy is highly infiltrative and is associated with a grim clinical outlook, elevated mortality, and a strong tendency to recur [2]. Standard treatment generally involves surgery followed by radiotherapy and chemotherapy with temozolomide (TMZ), a combined approach known as the Stupp protocol, which has remained the cornerstone of glioblastoma therapy for approximately twenty years [3]. Significant resistance to existing drugs and poor sensitivity to apoptosis‐inducing signals continue to undermine the effectiveness of current treatments [4]. Consequently, identifying novel chemotherapeutic agents and developing improved therapeutic approaches are essential for advancing glioblastoma management. One of the major hurdles in this pursuit is ensuring that promising therapies can be precisely targeted and successfully transported to tumor sites, overcoming the challenges imposed by the blood–brain barrier (BBB) to achieve adequate drug distribution within brain tumor tissue [5]. The BBB functions as a structural and biochemical shield, limiting molecular exchange between the circulatory system and the central nervous system. As a result, glioblastoma tumors are largely resistant to standard chemotherapeutic agents [6]. Endothelial cells of the BBB display high levels of several receptor systems, notably transferrin receptor 1 (TfR1), the insulin receptor, and the low‐density lipoprotein receptor (LRP1). LF is an approximately 80 kDa glycoprotein with the ability to bind iron. It can interact with LRP1, which facilitates its transport across the BBB [7]. Moreover, studies have indicated that LRP1 is present at elevated levels in glioblastoma cells [8], suggesting that LF could be utilized to specifically target these tumor cells. In the research conducted by Gai and colleagues, a variety of vanadium complexes were first synthesized, which were subsequently encapsulated into a LF‐V4 nanoparticle (NP) delivery system. This nanoplatform was able to penetrate the BBB by interacting with LRP1 and demonstrated selective targeting of glioblastoma, effectively suppressing the progression of glioblastoma tumors [9].

A multifunctional biomimetic nanoplatform termed L‐D‐I/NPs was developed by self‐assembling LF with the antimalarial drug dihydroartemisinin (DHA) and the photosensitizer indocyanine green. This engineered nanoplatform demonstrates targeted delivery to glioblastoma cells by interacting with LRP1 and efficiently crossing the BBB. Once internalized, it triggers ferroptosis in glioblastoma cells through the enhancement of intracellular reactive oxygen species levels and iron accumulation [10].

This study aims to elucidate the distinctive characteristics of LF and highlight critical factors in the design of novel LF‐based nanomedicine platforms capable of crossing the BBB, offering a promising approach for the effective treatment of glioblastoma.

In this manuscript that lactoferrin possesses several unique characteristics that distinguish it from other BBB‐targeting ligands commonly investigated for glioblastoma drug delivery, such as transferrin, angiopep‐2, insulin receptor ligands, and RVG peptides. Unlike many conventional targeting ligands that primarily function as transport mediators, lactoferrin exhibits intrinsic biological activities including antitumor, anti‐inflammatory, antioxidant, and immunomodulatory effects, which may provide additional therapeutic benefits beyond BBB transcytosis alone. Furthermore, lactoferrin can interact with low‐density lipoprotein receptor‐related protein‐1 (LRP1) and lactoferrin receptors, both of which are expressed on BBB endothelial cells and glioblastoma cells, thereby enabling dual‐targeting capacity. Compared with several peptide‐based ligands, LF also demonstrates favorable biocompatibility, relatively low immunogenicity, and the ability to serve simultaneously as both a targeting ligand and a bioactive therapeutic component.

## 2 Glioma

Neoplasms of the central nervous system develop from diverse cellular origins and account for approximately 2% of all cancer cases. The vast majority of malignant central nervous system tumors (approximately 95%) are located in the brain, and of these, approximately three‐quarters originate from glial cells, commonly referred to as gliomas [11]. Adult diffuse gliomas represent the most frequently occurring malignant tumors in the central nervous system. Patient survival differs substantially on the basis of the specific glioma subtype: low‐grade gliomas can have 5‐year survival rates of up to 80%, whereas high‐grade gliomas typically have 5‐year survival rates of < 5%. Despite differences in severity and prognosis, all gliomas exhibit aggressive infiltration and considerable resistance to treatment, making them effectively incurable [12].

### 2.1 Classification of Glioma

In recent years, molecular indicators have become increasingly central to diagnosing and categorizing gliomas, largely because they offer valuable insight into tumor behavior and patient outcomes. The World Health Organization (WHO) first emphasized the inclusion of molecular marker analysis in the evaluation of specific central nervous system tumors [13]. In the updated 2021 edition, the WHO broadened this guidance even further by adding additional biomarkers to the diagnostic framework. The 2021 update of the WHO classification system recognizes only three forms of diffuse gliomas in adults: astrocytomas characterized by mutations in isocitrate dehydrogenase (IDH), oligodendrogliomas that also harbor IDH mutations, and glioblastoma multiforme defined by the absence of IDH mutations (IDH–wild type) [14, 15].

### 2.2 Glioblastoma Multiforme

GBM is the most aggressive form of malignant brain cancer and is characterized by a high incidence and extremely poor patient survival. It represents approximately 14.7% of all tumors in the central nervous system and approximately 56.5% of all glioma cases [16]. The United States reports an overall incidence of 4.23%, whereas the rate among Asian and Pacific Islander populations is notably lower at 2.00% [17]. Because glioblastoma is highly aggressive and prone to return, its outlook is generally poor. Among all GBM patients, the median survival is approximately one year, and only approximately 21% survive for two years, whereas approximately 14% live for five years [18]. Glioblastoma can develop at virtually any age, although it is diagnosed most frequently in individuals between approximately 50 and 70 years of age. Data from the American Association of Neurological Surgeons indicate that the typical age at diagnosis is approximately 64 years. The disease also tends to affect men more often than women do [19].

The classical approaches for managing GBM primarily involve surgery followed by chemotherapy and radiation, which remain standard clinical practices. After tumor removal, temozolomide (TMZ) is the chemotherapy agent most frequently administered; it exerts its antitumor effects by adding methyl groups to purine bases in cancer cell DNA, leading to direct cellular damage [20]. TMZ exerts its primary cell‐killing effect by generating 6‐methylguanine DNA adducts, which in turn trigger tumor cell death through apoptosis, promote autophagic processes, and drive cells into senescence [21]. The ability of a drug to sensitize cells to radiation enhances the destruction of cancer cells when it is used alongside radiotherapy. However, treatment with TMZ is commonly associated with significant adverse effects, particularly blood‐related toxicity and reduced platelet counts, which have been observed in approximately 10%–20% of participants in phase II clinical studies [22].

**FIGURE 1:**
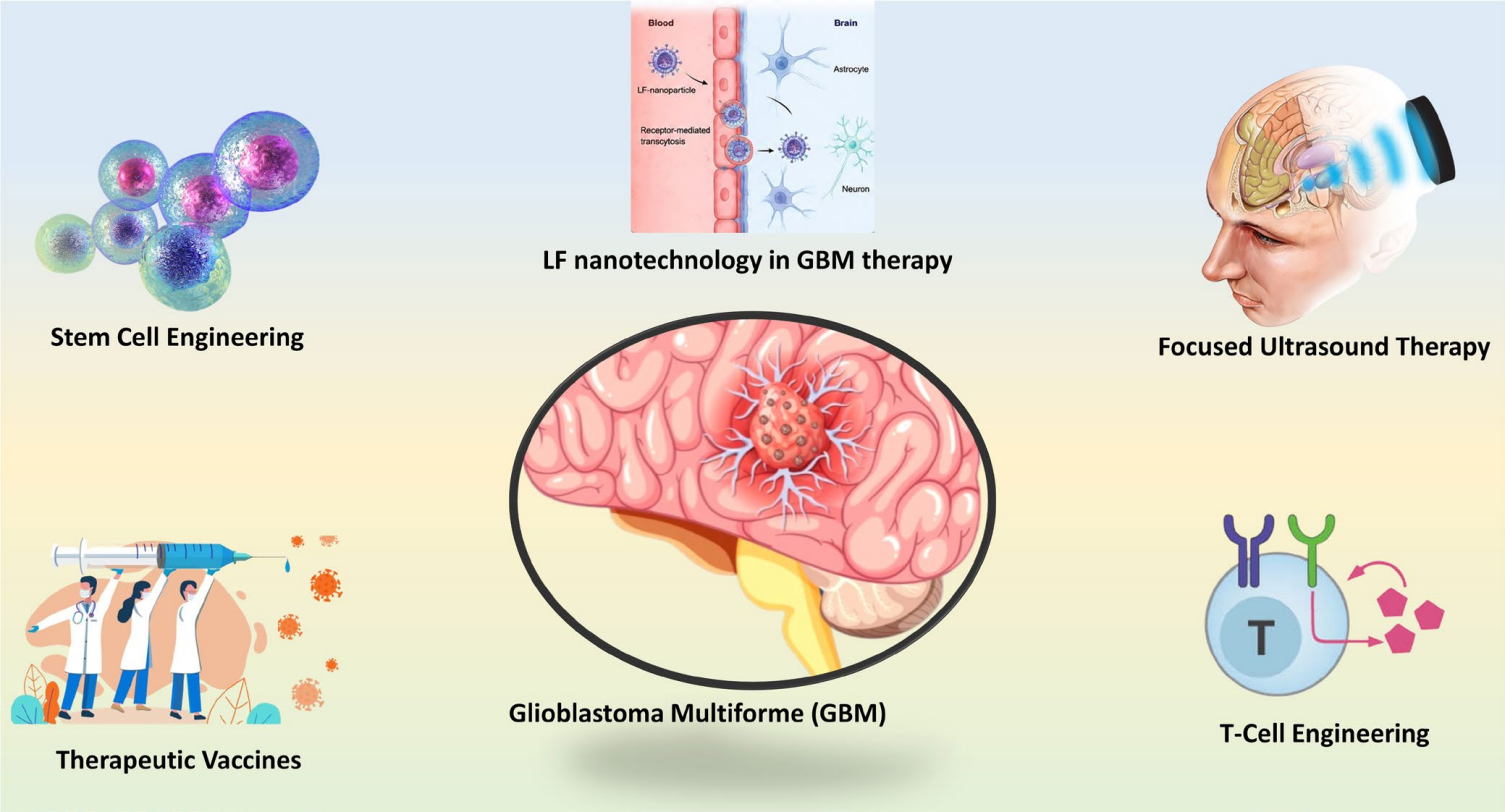
Novel glioblastoma therapies.

Current first‐line treatments for GBM frequently fail to prevent tumor relapse. Gaining a clearer picture of how glioma stem cells (GSCs) contribute to drug resistance along with the molecular programs that fuel their malignant transformation and tumor‐initiating capacity is essential for advancing therapeutic strategies. Similarly, a more detailed understanding of the architecture, physiology, and barrier functions of the BBB/BTB (Blood‐Tumor Barrier) will be key to designing new delivery platforms or refining existing platforms to improve the transport of anticancer agents. Emerging modalities, such as stem‐cell‐based therapies, polymeric delivery systems, engineered T cells, therapeutic vaccines, and focused ultrasound (FUS), are promising for enhancing drug delivery and promoting penetration across the BBB. As these innovative technologies continue to evolve, they hold the potential to drive meaningful progress for individuals facing this aggressive and currently incurable cancer [23] (Figure 1).

## 3 Lactoferrin

**FIGURE 2:**
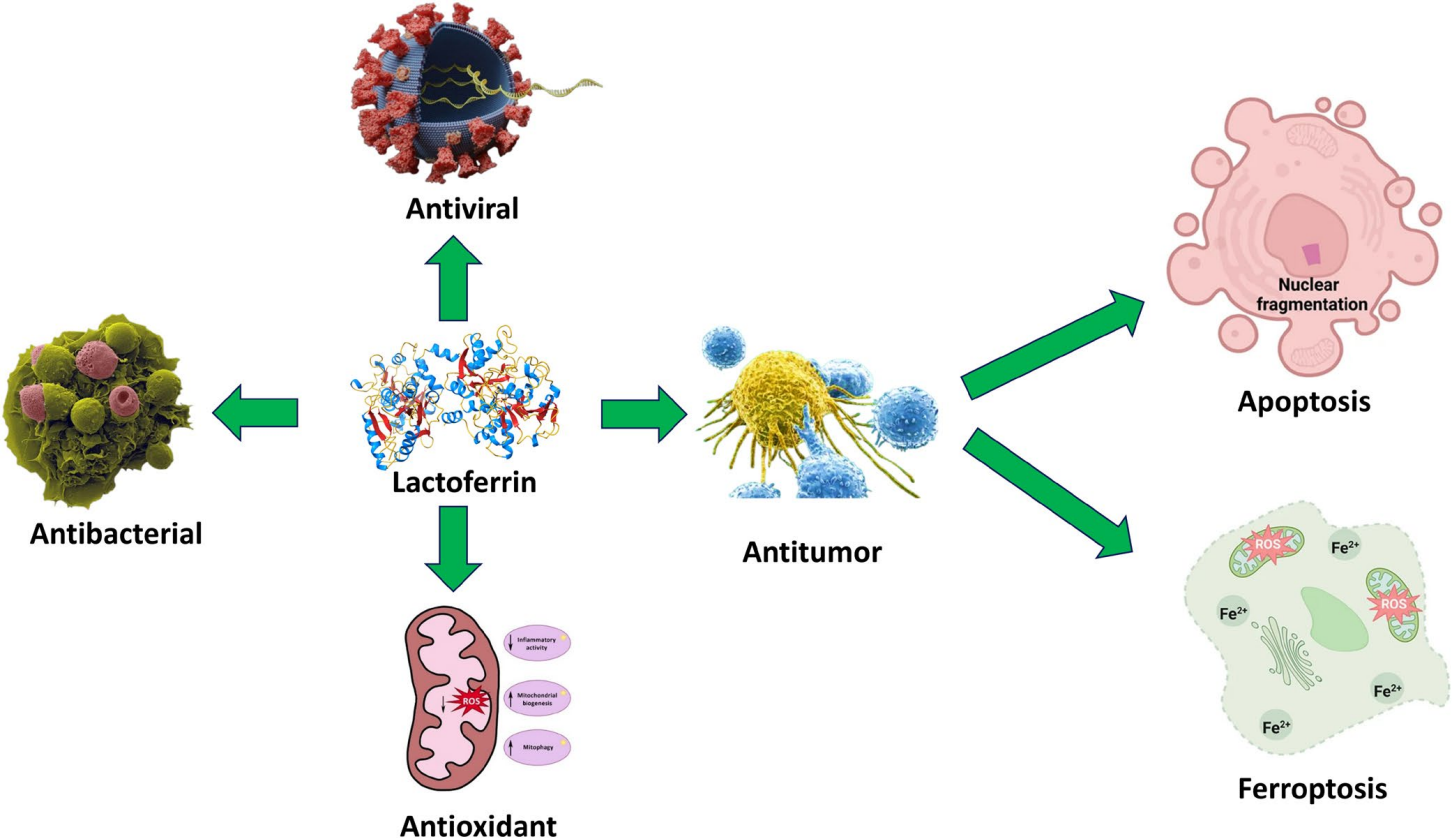
Diverse biological activities of LF.

LF is a glycoprotein that binds iron but does not contain heme, and it is a member of the transferrin protein family. It is present in a variety of exocrine secretions, such as breast milk, tears, bronchial mucus, and digestive fluids, and constitutes an important component of both human and bovine milk [24]. LF is discharged from the granules of activated neutrophils, so its levels in both blood plasma and stool increase when infections or inflammatory processes occur, reflecting the increased influx of neutrophils to affected sites [25]. Human lactoferrin (hLF) closely resembles bovine lactoferrin (bLF) in both its structure and biological role, although the levels of LF are markedly greater in human milk than in bovine milk [26]. LF is a key component of the body's protective system, contributing to defense mechanisms through its broad range of biological actions, such as inhibiting viruses and microbes, reducing oxidative stress, and regulating immune responses [27] (Figure 2). Emerging research indicates that LF may help defend against widespread viral infections by increasing type I interferon production, strengthening natural killer (NK) cell functions, and promoting cytokine responses associated with Th1‐type T‐helper cells [28].

Notably, delivery of hLF into the peritoneal cavity suppressed the expansion of primary tumors as well as the formation of metastatic lesions in the lungs, and this effect occurred regardless of the amount of iron carried by the protein. The same study also linked LF's antitumor activity to its capacity to stimulate NK cell responses [29]. The primary challenge in effectively treating GBM and the high likelihood of its recurrence stem from the inability of chemotherapy drugs to efficiently cross the blood–brain barrier and reach tumor cells [30]. The transport of therapeutic agents into the brain can be significantly improved through receptor‐mediated endocytosis or transcytosis at the BBB. Frequently investigated targets on the luminal surface of the BBB include the transferrin receptor, scavenger receptor class B type 1, lactoferrin receptor (LFR), and leptin receptor, as they facilitate the uptake and transport of molecules into the central nervous system. This approach has therefore drawn considerable scientific attention [31].

Currently, fluorescence imaging provides high sensitivity, noninvasiveness, and rapid analysis, making it an attractive approach for diagnosing Alzheimer's disease (AD). Owing to their low background signal, aggregation‐induced emission (AIE) probes have been widely explored for the detection of A*β* aggregates [32]. Zhu et al. synthesized four AIE fluorescent probes with distinct emission wavelengths. To enhance brain uptake of the fluorescent photosensitizer, TPE‐yne‐Indo was further encapsulated within the pores of mesoporous silica nanoparticles, forming a nanocarrier suitable for in vivo delivery. LF, as a ligand of the LFR, was also encapsulated in mesoporous silica nanoparticles to facilitate receptor‐mediated endocytosis on the surface of glial cells and the BBB crossing process. This multifunctional nanoplatform has shown great potential for the early diagnosis and treatment of AD [33].

### 3.1 Anti‐Inflammatory and Immunomodulatory Activities

LF exhibits significant immunomodulatory and anti‐inflammatory activities [34]. Its anti‐inflammatory action is linked to its ability to enter host cells via receptor‐mediated endocytosis and subsequently localize to the nucleus, where it can influence the transcription of genes involved in inflammation [35]. By downregulating proinflammatory cytokines, LF not only dampens inflammatory responses but also supports the adaptive arm of the immune system. In addition, LF has demonstrated effectiveness in mitigating and correcting iron‐related abnormalities. It achieves this by modulating immune activity and reducing the levels of cytokines such as Interleukin 6 (IL‐6), a finding consistently observed in both in vitro [36] and in vivo [37] models, as well as in clinical trials [38].

### 3.2 Application in Cancer Therapy

**FIGURE 3:**
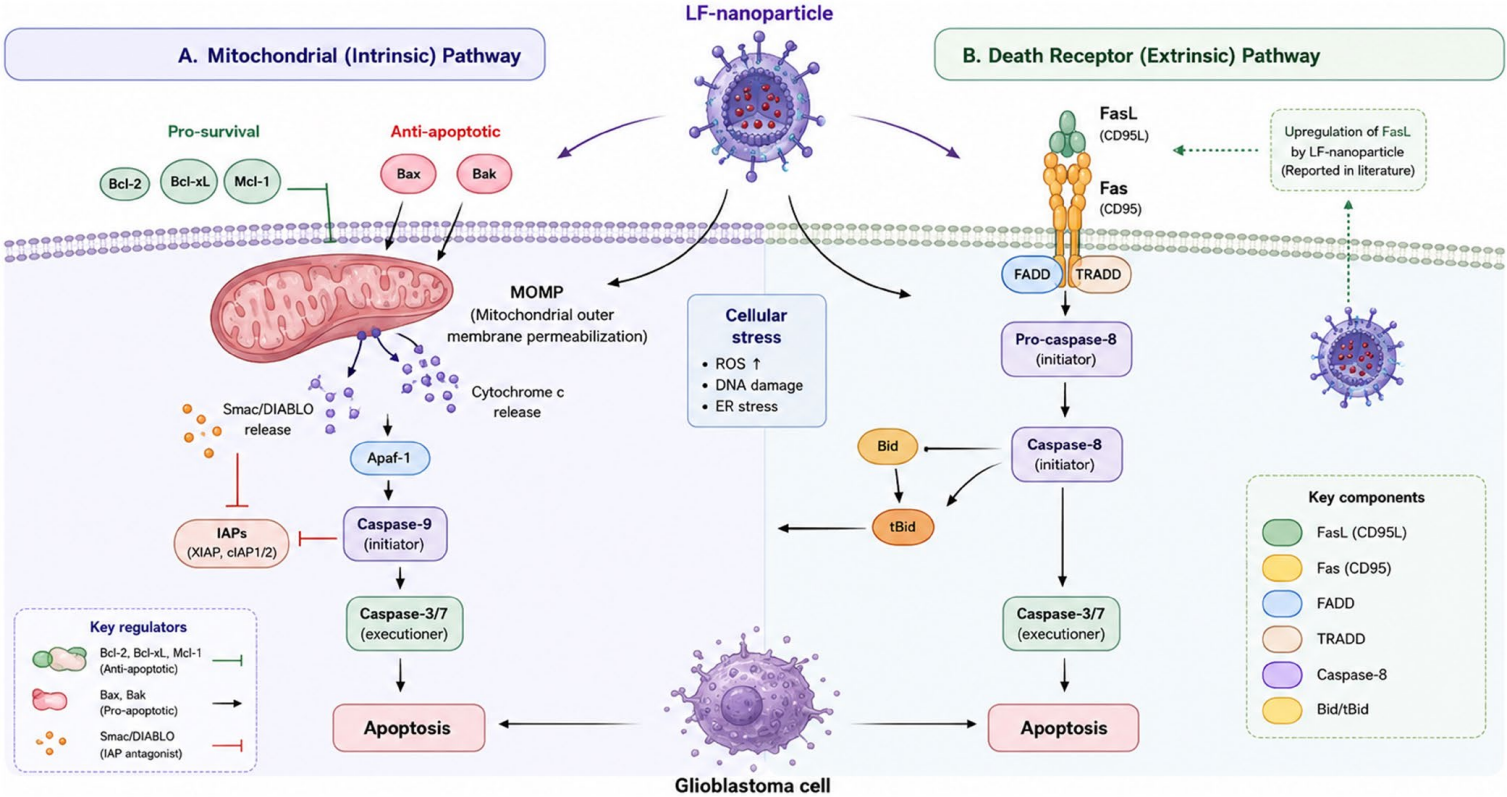
Schematic illustration of lactoferrin (LF) nanoparticle–induced apoptosis pathways in cancer cells. LF nanoparticles activate both mitochondrial (intrinsic) and death receptor (extrinsic) apoptotic pathways through modulation of Bcl‐2/Bax balance, cytochrome c release, Fas/FasL signaling, and caspase activation, ultimately leading to glioblastoma cell death.

Alterations in the LF gene within cells have been linked to a heightened risk of developing cancer. Administering LF or its modified forms from external sources has been shown to effectively suppress tumor progression and lower cancer vulnerability [39]. Various investigations have demonstrated that LF exerts cytotoxic effects on multiple cancer types through mechanisms such as disrupting cellular membranes, triggering apoptosis, and halting cell cycle progression (Table 1). More specifically, because LF possesses a relatively high isoelectric point (approximately 8.0–8.5), it tends to carry a positive charge under physiological conditions, resulting in a strong propensity to interact with the negatively charged membranes of cancer cells (Figure 3). The biological functions of LF arise through its ability to bind to various cellular receptors, such as CD14, CXCR4, heparan sulfate proteoglycans (HSPGs), intelectin‐1 (omentin‐1), LRP‐1/CD91, and the Toll‐like receptors TLR2 and TLR4 [91]. These receptor interactions provide opportunities to use LF as a targeting ligand. Because many of these receptors are overexpressed on the surface of cancer cells, they can promote receptor‐mediated endocytosis of LF‐modified delivery systems, thereby improving the internalization and transport of therapeutic agents [92].

**TABLE 1:** Nanoparticles and LF as anticancer agents.

Cancer	Mechanisms	Model	Dose	Cell line	Refs.
Liver leukemia	Apoptosis	In vitro		HepG2 Jurkat leukemia	[40]
Breast	Apoptosis	In vitro		4T1	[41]
Colon	Apoptosis	In vitro and in vivo	250 mg/kg	HT29	[42]
Breast	Immune suppressive and non‐immunological	In vitro and in vivo	1 mg/kg/day	4T1‐Luc	[43]
Cervical	Apoptosis	In vitro		HeLa, SiHa, KLE and HEC‐1A	[44]
Breast	Migration and invasion	In vitro		MCF‐7 and MDA‐MB‐231	[45]
Prostate	Necrosis and apoptosis	In vitro and in vivo	10 mg/kg	A549 and Mat Ly Lu	[46]
Breast	Apoptosis and necrosis	In vitro and in vivo	13 mg/kg/day	MCF‐7 4T1	[47]
Gastric	Apoptosis	In vitro		AGS	[48]
Prostate/Osteosarcoma	Apoptosis	In vitro		PC‐3 MG‐63 MDA‐MB‐231	[49]
Breast/Laryngeal	Apoptosis	In vitro		MDA‐MB‐231 HEp‐2	[50]
Prostate	Apoptosis	In vitro		PC3	[51]
Prostate		In vitro		PC‐3	[52]
Retinoblastoma		In vitro		Y79	[53]
Breast	Apoptosis	In vitro		MCF7	[54]
Breast	Apoptosis	In vitro and in vivo	2.5 mg/kg	MCF‐7	[55]
Breast	Ferroptosis	In vitro and In Vivo	0.6 mg/10 g	MDA‐MB‐231 MCF7	[56]
Colorectal	Apoptosis, autophagy and antiangiogenesis	In vitro and in vivo	1 mg/kg	CT26/HUVEC	[57]
Breast	Apoptosis	In vitro		MDA‐MB‐231/MCF‐7	[58]
Colorectal/liver/breast/prostate	Apoptosis	In vitro		Caco‐2/HepG‐2/MCF‐7/PC‐3	[59]
Breast	Apoptosis	In vitro and in vivo	2.5 PMT mg/kg	MDA‐MB‐231/BCE/A549	[60]
Breast/prostate/osteosarcoma	Apoptosis	In vitro		MDA‐MB‐231/Hs 578T/PC‐3/MG‐63/MCF‐10‐2A/T‐47D	[61]
Breast	Apoptosis	In vitro and in vivo	2 μmol/kg	MCF‐7/4T1	[62]
Breast		In vitro		MCF‐7	[63]
Breast	Apoptosis	In vitro		MDA‐MB‐231/MCF‐7	[64]
Prostate		In vitro		PC‐3‐Luc	[65]
Colorectal	Apoptosis	In vitro		HT‐29	[66]
Prostate	Apoptosis	In vitro		DU‐145	[67]
Prostate	Apoptosis	In vitro and in vivo	15 mg/kg	DU145	[68]
Breast	Apoptosis	In vitro and in vivo	5 mg/kg	4T1	[69]
Breast	Necrosis and Apoptosis	In vitro and in vivo			[70]
Colon		In vivo	10 mg/kg		[71]
Breast	Apoptosis and Necrosis	In vitro and in vivo	10 mg/kg	MDA‐MB‐231	[72]
Stomach	Apoptosis	In vitro		SGC‐7901	[73]
Breast	Apoptosis	In vitro		MDA‐MB‐231	[74]
Lung		In vitro and in vivo		A549	[75]
Breast	Apoptosis	In vitro		MCF‐7	[76]
Breast	Apoptosis	In vitro and in vivo	12 g	MDA‐MB‐231	[77]
Pancreatic	Apoptosis	In vitro		PANC‐1	[78]
Colon	Apoptosis	In vitro and in vivo	12 mg/kg	Caco‐2	[79]
Breast/Liver/Colon	Apoptosis	In vitro		MCF‐7/HepG2/Caco‐2	[80]
Breast	Apoptosis	In vitro		HS578T/T47D	[81]
Gastric	Apoptosis	In vitro		AGS	[82]
Breast	Apoptosis	In vitro and in vivo		MDA‐MB‐231	[83]
Colorectal		In vitro and in vivo	500 mg/kg/day 1000 mg/kg/day	RKO/RCN‐9	[84]
Breast	Apoptosis	In vitro		Hs 578T/T‐47D/MCF‐10‐2A/MDA‐MB‐231	[85]
Lung		In vitro		PC‐14/PC‐3	[86]
Breast	Apoptosis	In vitro and in vivo	5.5 mg/kg	MCF‐7/MDA‐MB‐231	[87]
Gastric		In vitro		AGS	[88]
Colon	Apoptosis			HCT116	[89]
Gastric	Apoptosis	In vitro		BGC‐823	[90]

*Note:* Due to substantial heterogeneity among in vitro experimental protocols, concentration ranges and exposure conditions are reported only when consistently available in the original studies.

Pterostilbene (PTS) has anti–breast cancer potential but suffers from low stability, solubility, and bioavailability. Aly et al. engineered solid lipid nanoparticles loaded with PTS (PTS‐SLNs) through ultrasonication. Their targeted delivery strategy involved modifying the nanoparticles with both LF and chondroitin sulfate (CS), producing dual‐functionalized CS/LF/PTS‐SLNs. This formulation more effectively modulated key molecular targets (VEGF, cyclin D1, caspase‐3, and BAX) and strongly reduced Bcl‐2 expression, demonstrating enhanced antitumor activity. CS/LF/PTS‐SLNs represent promising targeted nanocarriers for breast cancer therapy via the use of plant‐derived compounds [72].

In 2023, Elmorshedy et al. developed nanoparticle‐in‐microparticle delivery systems (NIMDs) for the oral delivery of docetaxel and ATR. By conjugating drugs onto a LF backbone and encapsulating them in calcium‐crosslinked microscale particles, NIMDs minimize drug release in the upper gastrointestinal tract while enabling sustained release in the colon. Compared with conventional nanoparticles, NIMDs more effectively inhibited NF‐κB, p‐AKT, and p‐ERK1/2 signaling in vivo, increasing their potential for use in CRC therapy [79]. In 2022, Yang and colleagues developed a metal‐based anticancer strategy using gallium‐derived C4 and LF‐C4 nanoparticles. Compared with individual components, these nanoparticles effectively target the TME, enhance tumor suppression, overcome codelivery challenges, and inhibit tumor angiogenesis while activating the immune response [62].

## 4 LF and Glioblastoma

HMGB1 (High‐Mobility Group Box‐1), which is released in the TME, drives tumor progression by promoting angiogenesis and growth. High HMGB1 levels are associated with advanced glioblastoma and poor prognosis. Glycyrrhizin (GL) can inhibit HMGB1 but suffers from poor pharmacokinetics and tumor delivery. The LF‐GLconjugate overcomes this challenge by targeting LFRs on the BBB and GBM cells, effectively suppressing HMGB1 in tumors. LF‐GL reduces angiogenesis and tumor growth, making it a promising strategy to counteract DAMP‐mediated tumor progression [93]. In glioblastoma, STAT3 is frequently hyperphosphorylated, a condition linked to poor patient prognosis. Cutone et al. investigated the impact of both native and iron‐saturated forms of bLF on the migratory behavior of human glioblastoma cell lines. Using a wound healing assay, they reported that bLF could partially or completely block cell migration, with the extent of inhibition depending on the protein's iron saturation level. At the molecular level, bLF reduced the expression of vimentin and SNAIL, upregulated cadherin, and suppressed the IL‐6/STAT3 signaling pathway. These findings suggest that bLF may represent a promising adjuvant strategy to complement conventional glioblastoma therapies [94]. Hwang developed a LF‐heparin conjugate that enables oral delivery and targeted anticancer therapy for glioblastoma. By utilizing the LFR, the conjugate can cross BBB‐like barriers, showing oral bioavailability and antitumor efficacy in a glioblastoma animal model [95]. Low‐density LRP1 is highly expressed on both brain capillary endothelial cells and glioblastoma cells, whereas its expression in normal brain parenchyma remains relatively limited. This differential expression profile has attracted considerable attention, as LRP1 can facilitate the transport of therapeutic agents across the blood–brain barrier (BBB), making it a promising molecular target for glioblastoma‐directed therapies. Among the ligands targeting this receptor, Angiopep‐2 (TFFYGGSRGKRNNFKTEEYC; ANG), a peptide derived from the Kunitz domain of aprotinin, exhibits strong affinity for LRP1 and has therefore emerged as an effective mediator for receptor‐targeted drug delivery to glioblastoma tissues [96].

He et al. developed an innovative pH‐responsive polymersome platform for glioblastoma treatment based on the self‐assembly of the amphiphilic copolymer polycaprolactone‐poly (2‐ethyl‐2‐oxazoline) (PCL‐PEOz). In this system, gold nanoparticles and doxorubicin were co‐encapsulated to establish a multifunctional therapeutic nanoplatform. Gold nanoparticles have been widely recognized as potent radiosensitizers capable of enhancing the efficacy of radiotherapy in glioblastoma, whereas doxorubicin is a well‐established anthracycline chemotherapeutic agent extensively used in solid tumor treatment. The simultaneous incorporation of these agents enabled the integration of radiotherapeutic and chemotherapeutic modalities within a single delivery system. In addition, encapsulation within polymersomes improved drug retention and reduced rapid systemic clearance by exploiting the enhanced permeability and retention (EPR) effect within tumor tissues. To further improve BBB penetration and tumor selectivity, the polymersomes were surface‐functionalized with Angiopep‐2. The resulting Angiopep‐2‐conjugated pH‐sensitive polymersomes (Au‐DOX@PO‐ANG) demonstrated enhanced BBB transcytosis and increased glioblastoma‐targeting capacity. In U87‐MG human glioblastoma xenograft models, this nanoplatform effectively combined radiotherapy and chemotherapy, leading to prolonged survival and improved therapeutic efficacy in nude mice [97]. Despite the promising role of LRP1‐mediated transport in LF‐based glioblastoma targeting, several limitations should be considered. LRP1 expression may vary across different GBM molecular subtypes, particularly between IDH‐mutant and IDH‐wildtype tumors, which could influence the efficacy of LF‐mediated delivery systems. Furthermore, intratumoral heterogeneity and patient‐specific factors such as age and tumor microenvironment may alter receptor expression and therapeutic responsiveness. These limitations highlight the need for personalized targeting strategies and further clinical validation of LF‐based nanocarriers.

### 4.1 Nanocarrier‐Based Lactoferrin Therapy for GBM

Both preclinical and clinical studies indicate that multiple factors can limit the effectiveness of therapeutic interventions, often resulting in suboptimal clinical outcomes. In the context of glioblastoma, the BBB represents a major obstacle. Recently, nanomaterials have emerged as promising tools to address these challenges, offering the potential to mitigate the unfavorable properties of therapeutic agents and enhance their performance. The BBB is estimated to prevent adequate brain delivery of nearly all therapeutics, with approximately 100% of large molecules and 98% of small molecules failing to reach effective concentrations in the brain. NPs can encapsulate these agents and be engineered to specifically target brain delivery or traverse the BBB, allowing access to tumors such as GBM that were previously difficult to treat. Depending on their composition, nanoformulations can carry both hydrophilic and hydrophobic drugs, provide controlled and sustained release, and prolong the circulating half‐life of therapeutics (Table 2) [118]. Zhang and colleagues developed a novel metal‐based therapeutic strategy for glioma by designing an Au complex (C2) that has strong cytotoxic effects on glioma cells. They further formulated LF‐C2 nanoparticles to enable this agent to cross the BBB, offering a promising new avenue for targeted glioma treatment. This work highlights an innovative approach for applying metal‐based compounds in precision therapies for brain tumors [98].

**TABLE 2:** Nanoparticles using LF as glioblastoma therapy.

Nanoparticles (NPs)	Mechanisms	Model	Dose	Cell line	Refs.
Au complex (LF‐C2 nanoparticles)	Apoptosis	In vitro and in vivo	5 μmol of Au/kg	U87MG	[98]
Silica	Apoptosis	In vitro, 2D and 3D models		U87	[99]
Oleylamine‐coated Fe_3_O_4_ magnetic NPs (OAM‐MNPs)		In vitro and in vivo	12 mg Fe/kg	Rat C6 glioma	[100]
Liposomes	Apoptosis	In vitro and in vivo	5 mg/kg	U87 MG	[101]
TMZ‐loaded LF NPs (TMZ‐LFNPs)	Apoptosis	In vitro and in vivo	10 mg/kg body weight	GL261, HepG2 and A549	[102]
Aurora Kinase B (AKB) siRNA‐loaded NPs (AKB–LFNPs)	Apoptosis	In vitro and in vivo	2 mg/kg	GL261	[103]
LF and muscone dual‐modified liposomes (LF‐LP‐Mu‐DTX)		In vitro and in vivo	5 mg/kg	U87‐MG	[104]
Rabies virus glycoprotein (RVG) and lactoferrin (LF)‐grafted liposomes (RGV‐LF‐liposomes)	Apoptosis	In vitro		U87 MG	[105]
SRL peptide‐coated dendrimer (PAMAM‐PEG‐SRL)		In vitro		C6 glioma	[106]
Cy5.5‐LF‐MPNA nanogels		In vitro and in vivo	12 mg Fe/kg body weight	C6 glioma	[107]
LF‐ and FA‐grafted PLGA NPs (LF/FA/PLGA NPs)	Necrosis and apoptosis	In vitro		U87MG	[108]
LF‐modified PEG‐PLGA NPs (LF‐NPs)		In vitro and in vivo	10 mg/kg	C6 glioma	[109]
LF conjugated iron oxide NPs (LF‐IONPs)		In vitro		C6 glioma	[110]
LF‐polymersome holding doxorubicin and tetrandrine (LF‐PO‐Dox/Tet)		In vitro and in vivo	0.5 mg/kg	C6 glioma	[111]
LF@GO@Fe_3_O_4_		In vitro		C6 glioma	[112]
Chitosan		In vitro		Glioma 261	[113]
Ultrasmall superparamagnetic iron oxide NPs (USPIONs)		In vitro and in vivo	10 mg Fe/kg	C6 glioma	[114]
LF‐conjugated superparamagnetic Iron oxide NPs (LF‐SPIONs)		In vitro and in vivo	12 mg Fe/kg	U251 and SHG‐44	[115]
Hyaluronic acid (HA) and chitosan Hydrochloride (CSH) (LF‐Cur‐PSNPs)		In vitro and in vivo	1.25 mg/kg	C6 glioma	[116]
LF‐DOX/PBNG		In vitro and in vivo	2.5 mg/kg	G422 cells	[117]

Transport proteins such as LF, transferrin, and albumin are highly expressed on the surface of the BBB, making them promising targets for the active and efficient transport of nanomedicines across the BBB and into tumors. LF, alongside other major BBB‐targeting ligands (transferrin, angiopep‐2, insulin receptor ligands, and RVG peptides), highlights their respective mechanisms, advantages, and limitations without claiming superiority. LF possesses a distinctive combination of properties (dual targeting, intrinsic antitumor activity, favorable biocompatibility) that makes it a promising and complementary ligand among the existing repertoire, not a replacement or superior option [6]. First, GBM cells and the BBB highly express LFRs, which cascade to target ligands. For the first time, Janjua and colleagues established a room‐temperature protocol for producing silica NPs characterized by ultrasmall size and large pores, allowing high‐capacity loading of chemotherapeutic agents as well as surface conjugation with targeting ligands. To achieve highly selective targeting, the nanoparticles were conjugated with LF. Doxorubicin, a potent chemotherapeutic that cannot cross the BBB naturally but shows strong in vitro activity against glioblastoma, was loaded onto these LF‐conjugated ultrasmall, large‐pore silica particles (USLP‐LF). Notably, USLP‐LF enhanced doxorubicin‐induced apoptosis in GBM cells in both the 2D and 3D models. Overall, this innovative nanoplatform has significant potential as a therapeutic strategy for GBM [99].

In a recent study, magnetic Fe_3_O_4_ nanoparticles were synthesized and coated with oleylamine (OAM‐MNPs) and then encapsulated within a novel amphiphilic copolymer composed of poly aminoethyl ethylene phosphate and poly l‐lactide (PAEEP‐PLLA). These OAM‐MNP‐loaded copolymer nanoparticles (M‐PAEEP‐PLLA‐NPs) were further functionalized with LF to enable targeted delivery specifically to glioma tumors. Both in vitro and in vivo MRI analyses demonstrated that LF‐M‐PAEEP‐PLLA‐NPs exhibit prolonged retention, enhanced tumor‐targeting capability, and significantly improved MRI contrast at the tumor site. This formulation provides a nanoscale MRI contrast agent designed for the precise targeting of brain gliomas [100]. Glioma cells and brain microvascular endothelial cells exhibit elevated levels of integrin αvβ3 and LFRs, suggesting opportunities for receptor‐specific therapeutic delivery. The RGD peptide interacts with αvβ3 integrin, promoting receptor activation and subsequent endocytosis, whereas LF assists in crossing the BBB and selectively targeting glioma cells. In this dual‐targeting approach, liposomes loaded with docetaxel and functionalized with both RGD and LF (RGD‐LF‐LP‐DTX) showed improved accumulation in the brain and superior antiglioma effects in experimental models [101].

Although LF‐based nanocarriers have demonstrated promising therapeutic efficacy in glioblastoma models, several important translational challenges remain unresolved. Direct quantitative comparison among different nanocarrier systems is difficult because of substantial heterogeneity in nanoparticle composition, surface modifications, therapeutic payloads, dosing protocols, experimental models, and outcome measurements across studies. In addition, important factors such as nanoparticle stability during systemic circulation, large‐scale manufacturing reproducibility, immunogenicity, long‐term biosafety, and batch‐to‐batch consistency continue to represent major barriers to clinical translation. While some studies reported enhanced BBB penetration and prolonged survival in preclinical glioblastoma models, others demonstrated only moderate improvements in therapeutic accumulation or limited targeting specificity, emphasizing the need for standardized evaluation frameworks and more clinically relevant experimental models. Future investigations should therefore focus on harmonizing experimental protocols and establishing comparative translational benchmarks for LF‐mediated nanotherapeutic systems [119]. In conclusion, critical discussion addressing how parameters such as nanoparticle size, surface charge (zeta potential), polydispersity index (PDI), and shape influence BBB penetration, cellular uptake, circulation half‐life, and biodistribution is necessary. Specifically, it notes that ultrasmall nanoparticles (< 50 nm) generally exhibit enhanced BBB transcytosis, whereas larger nanoparticles (> 100 nm) may be preferentially taken up by reticuloendothelial system organs. Similarly, it discusses how near‐neutral or slightly positive surface charges optimize receptor‐mediated transcytosis while avoiding nonspecific adsorption.

Also, this manuscript incorporates discussion of critical parameters including encapsulation efficiency, drug loading content, and release profiles (e.g., sustained vs. stimuli‐responsive release). It highlights that many LF‐based nanocarriers lack systematic optimization of these parameters, leading to batch‐to‐batch variability and suboptimal therapeutic performance. Controlled release mechanisms, including pH‐sensitive, redox‐sensitive, and enzyme‐responsive designs, are now critically evaluated in the context of the GBM tumor microenvironment. A critical gap identified is that most studies do not report the density of LF conjugated on nanoparticle surfaces (e.g., number of LF molecules per nanoparticle or ligand density per nm^2^). This parameter is crucial because insufficient ligand density results in poor targeting, whereas excessive density may cause steric hindrance, reduced receptor binding affinity, and altered pharmacokinetics. Standardized reporting of ligand density should be encouraged in future studies, as this parameter critically influences receptor‐mediated targeting efficiency, pharmacokinetics, and therapeutic performance. Although LF itself exhibits relatively low immunogenicity, its conjugation with synthetic nanomaterials, crosslinkers, or polyethylene glycol may induce immune‐related responses, including accelerated blood clearance following repeated administration and the development of anti‐PEG antibodies. These potential immunotoxicological concerns have received limited attention in the current literature and should be systematically evaluated during the preclinical and translational development of LF‐based nanocarriers.

During the past decade, nanotechnology has become a major driving force in the development of innovative therapeutic approaches, particularly through targeted drug delivery, immune regulation, and multifunctional therapeutic platforms. The nano‐based delivery systems investigated for glioblastoma therapy are summarized in Table 1. As highlighted throughout this review, nanomedicine offers considerable potential to overcome many of the biological and pharmacokinetic barriers that limit the efficacy of conventional GBM treatments. Nevertheless, significant obstacles still hinder successful clinical translation. The highly heterogeneous and adaptive nature of glioblastoma necessitates nanotherapeutic platforms capable of responding to continuously changing molecular and cellular tumor characteristics. In parallel, concerns regarding long‐term biosafety, immunogenic responses, large‐scale production, and manufacturing reproducibility remain important limitations for the broader clinical implementation of nanosystems. Furthermore, the discrepancy between encouraging preclinical findings and relatively limited clinical success emphasizes the need for more reliable translational models and standardized evaluation strategies. Future progress in this field will depend on the development of more sophisticated and biologically responsive nanosystems guided by multi‐omics profiling and tumor evolution dynamics. Equally critical is the expansion of well‐designed clinical studies that assess not only therapeutic efficacy but also safety, delivery efficiency, and regulatory feasibility. Through sustained interdisciplinary collaboration, nanomedicine may substantially advance glioblastoma treatment strategies and contribute to improved patient survival and clinical outcomes [120].

In a recent study, Liu et al. developed a silymarin‐repurposed and brain‐targeted photochemotherapeutic nanoplatform designed to enhance glioblastoma immunotherapy through synergistic mitochondrial suppression. This multifunctional system was generated through the self‐assembly of lactoferrin (LF), triphenylphosphine‐modified chlorin e6 (TCe6), and silymarin. Mechanistically, LF promoted selective targeting through interaction with low‐density lipoprotein receptor‐related protein‐1 (LRP1), thereby facilitating efficient blood–brain barrier penetration and glioblastoma accumulation. Following cellular uptake, TCe6‐induced reactive oxygen species (ROS) generation together with silymarin‐mediated glycolytic inhibition triggered mitochondrial dysfunction in GBM cells. These alterations subsequently activated the AMPK signaling pathway, resulting in programmed death‐ligand 1 (PD‐L1) degradation and stimulation of the cGAS–STING signaling cascade, ultimately enhancing antitumor immune activity.

Notably, this LF‐based nanoplatform significantly improved BBB permeability and potentiated antitumor immunity, representing a promising drug‐repurposing strategy for glioblastoma immunotherapy. The developed LS‐TCe6/NPs effectively enhanced intracellular ROS accumulation and induced tumor cell death through mitochondria‐targeted mechanisms. In addition, suppression of PD‐L1 expression and reduction of regulatory T‐cell (Treg) populations contributed to immune checkpoint modulation. Concurrently, activation of the cGAS–STING pathway together with photodynamic therapy‐induced immunogenic cell death promoted dendritic cell activation and increased T‐cell infiltration. In vivo studies further demonstrated that LS‐TCe6/NPs inhibited glioblastoma progression, prolonged survival, and exhibited favorable biosafety and biodistribution profiles. These findings highlight the growing potential of LF‐based nanocarriers not only as drug delivery systems but also as modulators of the glioblastoma tumor microenvironment and antitumor immunity [121].

## 5 Future Applications

On the basis of the capabilities of LF and advancements in nanocarrier technologies, several promising future applications can be anticipated for improving GBM treatment: LF's natural ability to cross the BBB and interact with cancer cell membranes positions it as an ideal targeting ligand. Future therapies may use LF‐modified nanocarriers to deliver chemotherapeutic agents, siRNAs, or gene‐editing tools directly into GBM tissue with enhanced precision. LF can simultaneously exert antitumor, anti‐inflammatory, and immunomodulatory effects. When combined with nanocarriers, future treatment regimens may integrate chemotherapy, immunotherapy, and gene therapy within a single platform to overcome the drug resistance and invasive nature of GBM. Because LF can accumulate in tumor tissue, it may be used as a target for imaging agents. Future applications include LF‐guided MRI contrast agents or fluorescent probes. Theranostic nanocarriers that combine imaging and treatment into one platform may enhance real‐time monitoring of therapeutic outcomes. Advances in molecular profiling may allow tailoring LF‐nanocarrier combinations to individual GBM subtype characteristics, improving treatment specificity and long‐term survival rates.

Building upon the current understanding of LF's biological functions and nanotechnology platforms, several promising yet underexplored directions can be identified for advancing LF‐based glioblastoma therapy. These future applications are organized below according to their technological maturity and potential clinical impact. Currently, LF‐based nanocarriers are applied to GBM without consideration of tumor molecular heterogeneity. Given that LRP1 and LFR expression levels vary significantly between IDH‐mutant and IDH‐wildtype GBMs, as well as across other molecular subtypes (e.g., mesenchymal, proneural, neural, classical), future studies should stratify patients based on receptor expression profiles. Preclinical evaluation of LF‐nanocarriers in patient‐derived xenograft (PDX) models representing distinct molecular subtypes will be essential to identify which GBM populations derive the greatest benefit. Ultimately, companion diagnostic imaging agents (e.g., LF‐conjugated PET or MRI tracers) could be developed to noninvasively assess LRP1/LFR expression levels prior to treatment. Recent evidence indicates that LF‐based nanoplatforms can modulate antitumor immunity through PD‐L1 degradation and cGAS‐STING activation [121]. Future iterations should intentionally combine LF‐mediated drug delivery with immune checkpoint inhibitors (e.g., anti‐PD‐1, anti‐CTLA‐4), CAR‐T cell therapies, or oncolytic viruses. Furthermore, LF's inherent ability to polarize tumor‐associated macrophages (TAMs) from an immunosuppressive M2 phenotype toward a pro‐inflammatory M1 phenotype remains underexplored in GBM. Designing LF‐nanocarriers that co‐deliver immunomodulatory agents (e.g., STING agonists, TLR agonists) alongside chemotherapeutics represents a high‐priority direction. The integration of diagnostic and therapeutic functions (theranostics) within a single LF‐based nanocarrier would enable real‐time monitoring of drug biodistribution, target engagement, and therapeutic response.

## 6 Conclusion

Even though today's standard treatments for glioblastoma, such as removing as much of the tumor as safely possible, radiation therapy, chemotherapy, and newer immune‐based therapies are useful, they still fall short of being perfect or final solutions. This gap exists because each of these treatments has built‐in limits and side effects, and together, they inevitably reduce patients' overall quality of life. Importantly, LF shows high biological uptake and notable selectivity toward tumor cells. On a mechanistic level, it influences a wide array of molecular pathways, allowing it to curb key cancer‐driving events at once, including uncontrolled cell growth, resistance to apoptosis, enhanced movement, infiltration into surrounding tissues, and the spread of cancer to distant sites. The diverse actions of LF are largely determined by the particular cellular context in which it operates, leading to distinctly different biological effects. Notably, LF displays a striking functional duality: in healthy cells, it enhances cell cycle progression and promotes proliferation, whereas in cancerous cells, it produces the opposite outcome, restricting growth by limiting motility and invasive behavior. In addition to its natural biological roles, LF can impede the development of cancer or slow the growth of existing tumors by markedly strengthening adaptive immune responses. This key antitumor activity combined with its exceptional capacity to cross the BBB positions LF as a vital therapeutic option for targeting tumors within the brain. Moreover, recent findings identifying LF as an efficient biological nanocarrier for delivering cytotoxic drugs have significantly increased its medical relevance. As a result, LF has emerged as a highly promising candidate for both preventing and treating GBM, especially when it is incorporated into combination therapy approaches.

## Author Contributions

Atena Abed, Javad Amini Mahabadi wrote the original draft and validated the manuscript. Javad Amini Mahabadi drew all figures. Atena Abed, Javad Amini Mahabadi conceptualized, reviewed, and edited the manuscript.

## Funding

The authors have nothing to report.

## Conflicts of Interest

The authors declare no conflicts of interest.

## Data Availability

The data and figures that support the findings of this study are available from the corresponding author, Javad Amini Mahabadi upon reasonable request.
